# Is xylem of angiosperm leaves less resistant to embolism than branches? Insights from microCT, hydraulics, and anatomy

**DOI:** 10.1093/jxb/ery321

**Published:** 2018-09-01

**Authors:** Matthias Klepsch, Ya Zhang, Martyna M Kotowska, Laurent J Lamarque, Markus Nolf, Bernhard Schuldt, José M Torres-Ruiz, De-Wen Qin, Brendan Choat, Sylvain Delzon, Christine Scoffoni, Kun-Fang Cao, Steven Jansen

**Affiliations:** 1 Institute of Systematic Botany and Ecology, Albert-Einstein-Allee 11, Ulm University, Ulm, Germany; 2 Department of Biological Sciences Faculty of Science, Macquarie University, NSW, Australia; 3 Plant Ecology, Albrecht von Haller Institute for Plant Sciences, University of Göttingen, Untere Karspüle, Göttingen, Germany; 4 BIOGECO, INRA, University of Bordeaux, Pessac, France; 5 EGFV, INRA, University of Bordeaux, Villenave d’Ornon, France; 6 Hawkesbury Institute for the Environment, University of Western Sydney, Richmond, New South Wales, Australia; 7 Université Clermont-Auvergne, INRA, PIAF, Clermont-Ferrand, France; 8 Guangxi Key Laboratory of Forest Ecology and Conservation, College of Forestry, Guangxi University, Daxuedonglu, Nanning, Guangxi, PR China; 9 Department of Biological Sciences, California State University, Los Angeles, State University Drive, Los Angeles, CA, USA

**Keywords:** Bordered pit, branch, embolism, hydraulic segmentation, leaf, microCT, pit membrane, xylem

## Abstract

According to the hydraulic vulnerability segmentation hypothesis, leaves are more vulnerable to decline of hydraulic conductivity than branches, but whether stem xylem is more embolism resistant than leaves remains unclear. Drought-induced embolism resistance of leaf xylem was investigated based on X-ray microcomputed tomography (microCT) for *Betula pendula*, *Laurus nobilis*, and *Liriodendron tulipifera*, excluding outside-xylem, and compared with hydraulic vulnerability curves for branch xylem. Moreover, bordered pit characters related to embolism resistance were investigated for both organs. Theoretical *P*_50_ values (i.e. the xylem pressure corresponding to 50% loss of hydraulic conductance) of leaves were generally within the same range as hydraulic *P*_50_ values of branches. *P*_50_ values of leaves were similar to branches for *L. tulipifera* (−2.01 versus −2.10 MPa, respectively), more negative for *B. pendula* (−2.87 versus −1.80 MPa), and less negative for *L. nobilis* (−6.4 versus −9.2 MPa). Despite more narrow conduits in leaves than branches, mean interconduit pit membrane thickness was similar in both organs, but significantly higher in leaves of *B. pendula* than in branches. This case study indicates that xylem shows a largely similar embolism resistance across leaves and branches, although differences both within and across organs may occur, suggesting interspecific variation with regard to the hydraulic vulnerability segmentation hypothesis.

## Teaser

A relatively high resistance of leaf xylem to embolism may not explain hydraulic segmentation between leaves and branches in angiosperms.

## Introduction

Ever since the pioneering experiments by 18th and 19th century botanists, it has been well known that leaves play a crucial role in long-distance water transport of plants (Hales, 1727; Böhme, 1893; Strasburger, 1893). As water transitions from liquid to vapour in leaf tissues, it generates an important pull, transmitted to the xylem water column through the cohesive strength of the liquid water. This results in a negative pressure in the hydraulic pathway from roots to stems and leaves (Tyree and Ewers, 1991; Buckley *et al.*, 2017). Although there is general agreement based on a large body of evidence for the cohesion–tension theory (Dixon, 1914; Scholander *et al.*, 1965; Dixon and Tyree, 1984; Holbrook *et al.*, 1995; Pockman *et al.*, 1995), an important, controversial question concerns the temporal and spatial frequency of embolism in xylem conduits along the entire xylem pathway at the whole-plant level (e.g. Jansen and Schenk, 2015; Venturas *et al.*, 2017).

Understanding how often and at what water potential xylem embolism formation occurs *in planta* is important for evaluating the risk of hydraulic failure, plant adaptations to drought, and future species distribution patterns (Sack *et al.*, 2016). Generally, the pressure required to induce 50% loss of xylem hydraulic conductivity (*P*_50_, MPa) is a useful trait in comparing embolism resistance across species and different organs (Choat *et al.*, 2012; Bouche *et al.*, 2016*b*). Given concerns about potential artefacts associated with manipulating a transport system under negative pressure (Jansen and Nardini, 2014; Torres-Ruiz *et al.*, 2014), various novel methods have been developed over the past years to improve the accuracy of the results, such as direct observation of embolism in conduits (Windt *et al.*, 2006; Brodribb *et al.*, 2016*b*; Choat *et al.*, 2016). The high resolution and optimal phase contrast obtained with X-ray computed tomography are especially useful to study the dynamic and temporal aspects of embolism occurrence and spread across xylem *in vivo* (Brodersen *et al.*, 2013; Choat *et al.*, 2015; Bouche *et al.*, 2016*b*; Scoffoni *et al.*, 2017*b*; Nolf *et al.*, 2017).

According to the initial interpretation of the hydraulic segmentation hypothesis, distal organs (e.g. leaves) in a tree become hydraulically isolated from the more proximal stems (e.g. main stem) due to the drop in water potential along the hydraulic continuum across organs, but with similar vulnerability to xylem embolism between distal and proximal segments (Zimmermann, 1983). This hypothesis, however, was later modified as the ‘hydraulic vulnerability segmentation’ hypothesis, suggesting that distal organs show a higher vulnerability to xylem embolism, which may contribute to the hydraulic safety of the proximal, more permanent, high-investment tissues of stems (Tyree and Ewers, 1991; Pivovaroff *et al.*, 2014; Johnson *et al.*, 2016; Wolfe *et al.*, 2016; Zhu *et al.*, 2016). Although a recent meta-analysis showed that leaves in 60 out of 73 species were more vulnerable to decline of hydraulic conductivity than stems (Scoffoni and Sack, 2017), it remains unclear whether or not this pattern is caused by leaves exhibiting more vulnerable xylem to embolism than branches or stems. Indeed, leaf hydraulic conductance (*K*_leaf_) quantifies the efficiency of water movement through both the leaf xylem and outside-xylem pathways, and recent studies have suggested that outside-xylem pathways could be especially vulnerable to dehydration (Trifiló *et al.*, 2016; Scoffoni *et al.*, 2017*a*).

Recent work based on direct observation of xylem embolism in leaf veins and branches within a single species showed a clear difference in vulnerability to embolism between distal and proximal organs in grapevine (Charrier *et al.*, 2016; Hochberg *et al.*, 2016), a difference between roots, stems, and leaves of olive (Rodriguez-Dominguez *et al.*, 2018), but a lack of segmentation in *Pinus pinaster* and tomato (Bouche *et al.*, 2016*a*; Skelton *et al.*, 2017). This may indicate that the hydraulic vulnerability segmentation hypothesis does not hold universally, or that hydraulic segmentation is not determined by a difference in xylem embolism resistance but by anatomical segmentation at the branch–petiole tissue level (André *et al.*, 1999) and/or the outside-xylem tissue (Trifiló *et al.*, 2016; Scoffoni *et al.*, 2017*a*).

Since xylem anatomy plays an important role in determining hydraulic safety (e.g. Lens *et al.*, 2011; Hacke, 2014; Jansen and Nardini, 2014), detailed anatomical observations using light and electron microscopy are highly appropriate to complement experimental measurements and observations. Drought-induced embolism is especially associated with the structure of bordered pits, which represent openings in the secondary cell walls between neighbouring vessels and tracheids (Tyree and Sperry, 1989; Choat *et al.*, 2008). In angiosperms, the thickness of intervessel pit membranes of freshly embedded wood samples was found to be closely associated with embolism resistance, although a functional, mechanistic understanding of this relationship remains unclear (Jansen *et al.*, 2009; Li *et al.*, 2016; Zhang *et al.*, 2017). While some attention has been paid to intra-tree variation of xylem in gymnosperms, especially with respect to conduit diameter and pit dimensions (Domec *et al.*, 2009; Bouche *et al.*, 2016*b*; Losso *et al.*, 2018), it is unclear if angiosperm pit membranes of leaf xylem show any significant difference in their ultrastructure and pit membrane thickness compared with branch xylem.

The aim of this study was to visualize embolism formation under different levels of drought stress in leaf veins (midrib, second and third vein order) of three angiosperm tree species using X-ray microcomputed tomography (microCT). Because earlier microCT observations showed leaves to be relatively strongly resistant to xylem embolism (Bouche *et al.*, 2016*a*; Scoffoni *et al.*, 2017*a*), we hypothesize that leaf and branch xylem will show similar resistances to embolism, contrary to what the hydraulic vulnerability segmentation hypothesis predicts. If leaves and branches were to show no substantial differences in their embolism resistance, pit membrane thickness may not differ between branches and leaves. Anatomical observations could thus provide additional support to test the vulnerability segmentation hypothesis between leaves and branches.

## Material and methods

### Leaf vulnerability to embolism

#### Plant material

Branches from two temperate (*Betula pendula* L. and *Liriodendron tulipifera* L.) and one Mediterranean (*Laurus nobilis* L.) angiosperm species were collected between 4 and 8 October 2016. All branches showed green leaves without autumn colours and were collected from a single specimen per species. For *B. pendula*, 16 branches were collected from a 5 m-tall tree growing at the Paul-Scherrer-Institute (Villingen, Switzerland). The 26 branches of *L. nobilis* studied came from a 1.5 m potted plant cultivated at the Botanical Garden of Ulm University, and 24 samples of *L. tulipifera* were from a 2 m-tall sapling bought at a local plant nursery in Brugg (Switzerland). Branches with a length of 30–70 cm were cut in air and immediately transferred to the laboratory at the TOMCAT beamline of the Swiss Light Source (SLS; Villigen, Switzerland). The cut branches had a diameter of *ca* 1 cm and included three to five growth rings. Samples were bench-dehydrated over 3–4 d to induce a wide range of xylem water potentials between −0.5 and −9.5 MPa.

Since all measurements were based on the same individual per species (*n*=1 specimen), intraspecific variation was excluded. At the same time, however, this approach indicated that the anatomical observations and quantification of xylem embolism were not completely independent from each other. Therefore, no generalization from a single specimen to the species level can be made.

#### X-ray microtomography

Leaves from cut branches were scanned at the TOMCAT beamline (X02DA) of the Swiss Light Source over a total of 72 h between 4 and 8 October 2016. The main advantage of working with cut branches was that these could be mounted on the beamline’s samples holder, while this was not possible for the intact trees. Moreover, cut branches dried relatively quickly and allowed us to perform scans over a wide range of xylem water potentials. Although working with cut branches may result in artificial xylem embolism in branches, especially near the cut end (Wheeler *et al.*, 2013; Torres-Ruiz *et al.*, 2014), a similar cutting artefact has not been found for leaves (Scoffoni and Sack, 2015). Moreover, recent work based on the optical method is not known to affect embolism resistance in leaves of cut branches (Brodribb et al., 2016a, b), while the presence of heterogeneous vessels (i.e. vessels characterized by helically thickened elements in their middle region, with homogeneously lignified thickenings on both vessel ends) in leaf abscission zones and stem–petiole transitions may function as safety devices along the water delivery pathway, contributing to hydraulic compartmentalization (André *et al.*, 1999; Rančić *et al.*, 2010).

The time period between branch excision, sample preparation, and microCT scanning was *ca* 10 min. Terminal branch ends with a length varying from 30 to 70 cm were mounted on an acrylic rod and placed on the beamline’s sample holder. Although the maximum vessel length in *L. nobilis* can be longer than the terminal branch ends used, vessels become shorter in terminal branch ends and are unlikely to run directly from the cut end into the distal leaves studied (André *et al.*, 1999; Choat *et al.*, 2016). Leaves and side branches were carefully fixed to the rod with tape to allow free rotation of the sample over 180°. Special care was taken not to damage the plant material during mounting. The scanned area included the midrib near the centre of the leaf with lamina in its vicinity that enclosed the second and third vein order. After taking a scan, branches were dried on a lab bench to conduct additional scans on other leaves from the same branch.

MicroCT scans were acquired with a synchrotron X-ray beam at 20 keV energy, with a current of 401 mA. A LuAG:Ce 20 µm scintillator, coupled with a sCMOS camera (pco.edge 5.5, PCO, Kelheim, Germany) was used to collect X-ray projections. Samples were slowly rotated from 0° to 180° using continuous rotation, while a total of 1501 2D images were obtained per scan. Images were acquired at 150 ms exposure time with 10-fold magnification and a field of view of 1.7 mm. 3D scan volumes with a voxel size of 0.65 µm were reconstructed with software developed at the SLS using simultaneous phase and amplitude extractions (Paganin *et al.*, 2002). Each scan took less than 2 min, reducing the possibility of considerable dehydration during the scanning period.

After the initial scan, the midrib was cut with a razor blade within the scan area while the sample was still attached to the acrylic rod on the beamline’s sample holder. This cutting induced locally (i.e. within 1 mm from the cut surface) 100% embolism of the functional, water-conducting conduits, which were now cut open. We then took a second scan of the leaf at exactly the same position (Supplementary Fig. S1 at *JXB* online), which was done within 5 min. Comparison of microCT slices before and after cutting the petiole allowed us to estimate the total number and cross-sectional area of all water conducting cells (including vessels and tracheids) within the midrib xylem that was scanned, as well as the relative amount of embolism before cutting the midrib. In most cases, corresponding microCT images were selected for this comparison, showing the same vessels, with a similar vessel arrangement before and after cutting (Supplementary Fig. S1). However, since the total number of conduits and the total conduit surface area was consistent within a vascular bundle over the 1.7 mm axial scan area, it was not necessary to select the most matching slices before and after cutting the midrib. Most importantly, the microCT slice that was selected after cutting the midrib was within 1 mm from the cut surface to make sure that all conduits were cut open and embolized.

Whenever possible, we tried to include second and third vein orders in the scanned area to compare embolism resistance between midribs and these veins (Supplementary Fig. S2). In case of *B. pendula*, 13 out of the 16 leaves scanned showed second and third veins, while this ratio was 21/26 and 20/24 for *L. nobilis* and *L. tulipifera*, respectively. Due to the reticulate venation pattern, it was practically not feasible to centre second and third vein orders within the 1.7 mm field of view over 180°. While second and third vein orders could easily be distinguished from the midrib, we were unable to identify in most cases the exact nature of the second or third veins within the 1.7 mm field of view.

Two to three leaves per branch that were located below the scanned leaf were wrapped in aluminium foil and enclosed in a water-vapour-saturated plastic bag for at least 1 h prior to measurements to allow leaf water potential to equilibrate with stem water potential. Before each microCT scan, leaf water potential was measured using a PMS Model 1000 pressure chamber (PMS Instruments, Albany, OR, USA).

All individual conduit areas (*A*_C_, µm^2^) were measured with ImageJ (version 1.48v, National Institutes of Health, Bethesda, MD, USA). The conduit area obtained was then used to predict the theoretical hydraulic conductance (*K*_TH_, kg s^−1^ MPa^−1^) based on the Hagen–Poiseuille equation, assuming a circular conduit shape (Tyree & Ewers, 1991):

$$K_{\text{TH}}=\frac{\pi \rho D^{4}}{128\eta }$$

where *D* is the conduit diameter corresponding to *A*_C_, ρ the density of water (0.9982 g cm^−3^), and η the viscosity of water (1.002 N s m^−2^). Adding up *K*_TH_ for all individual conduits within a vascular bundle provided the overall, theoretical conductance of this vein. The ratio between the theoretical maximum xylem conductance based on microCT after cutting (*K*_THmax_) with all conduits embolized, and the initial conductance prior to cutting (e.g. initial degree of embolism in the leaf; *K*_THinitial_) was equal to the theoretical loss of hydraulic conductance ( ${\text{PLC}}_{K_{\text{TH}}}$).

$${\text{PLC}}_{K_{\text{TH}}}=\frac{K_{\text{THinitial}}}{K_{\text{THmax}}}\times 100$$

The theoretical loss of hydraulic conductance was plotted versus the corresponding water potential values. The theoretical percentage loss of conductance ( ${\text{PLC}}_{K_{\text{TH}}}$) was fitted to a sigmoidal function with the following equation using SigmaPlot version 12.5 (Systat Software Inc.):

$${\text{PLC}}_{K_{\text{TH}}}=\frac{100}{1+\text{exp}\left(\left(\frac{S}{25}\right)\left(\Psi -b\right)\right)}$$

where *S* is the slope of the curve, and *b* is the *P*_50_ (Pammenter & Van der Willigen, 1998).

### Branch vulnerability to embolism

Branch vulnerability to embolism based on samples from the same trees was estimated using the centrifuge-flow technique. Unlike leaves, hydraulic measurements of branch xylem reflect xylem processes only. Moreover, microCT of cut branches might cause air entry into the branch xylem, depending on the vessel length, which could lead to underestimation of embolism resistance (Torres-Ruiz *et al.*, 2014). Although the application of two different methods to estimate embolism resistance between branches and petioles requires some caution, there is convincing evidence and strong agreement between the theoretical *P*_50_ values based on microCT and hydraulic *P*_50_ values based on centrifuge-based vulnerability curves for various species with tracheids and relatively short vessels (Choat *et al.*, 2010, 2016; Bouche *et al.*, 2016*b*; Scoffoni *et al.*, 2017*b*; Torres-Ruiz *et al.*, 2017; Charrier *et al.*, 2018; Lamarque *et al.*, 2018). Moreover, branches from intact plants could not be scanned because of the relatively tall size of the specimens selected (from 1.5 to 5 m).

A the end of the synchrotron campaign, six branches of *B. pendula* and four branches of *L. tulipifera* were cut, wrapped in wet paper tissue and plastic bags, and transported to Göttingen University for measuring embolism resistance of the branch xylem. Since all branches were collected from the same individuals, they had a similar age and size as the ones used for microCT. The centrifuge flow technique allowed us to compare leaves and branches from exactly the same plant material at the same time, avoiding any potential variation due to intraspecific difference, developmental differences, and/or seasonal variation.

The centrifuge flow technique (cavitron) with a standard (30 cm) rotor was applied to branches of *B. pendula* and *L. tulipifera*, which had a maximum vessel length of 23.5 ± 4.0 and 17.4 ± 1.6 cm (mean ±SE), respectively, based on air injection (*n*=5 per species). Samples were mounted in a custom-built rotor chamber in a modified centrifuge (Sorvall RC-5C, Thermo Fisher Scientific, Waltham, MA, USA), and spun at various velocities. The initial pressure applied to the sample was −0.58 MPa and was stepwise increased until the percentage loss of hydraulic conductivity (PLC, %) reached at least 90% (Cochard *et al.*, 2005). For each individual branch segment, the relationship between the applied pressure and PLC was plotted in a vulnerability curve. The *P*_50_ value (MPa) was calculated as the mean of the individual measurements per species.

We did not apply the 30 cm-long rotor to *Laurus nobilis* due to concerns about artefacts with this technique for species that have vessels exceeding 28 cm in length (Choat *et al.*, 2010; Wang *et al.*, 2014; Torres-Ruiz *et al.*, 2017). Instead, branch xylem embolism resistance of this species was based on Lamarque *et al.* (2018) using centrifuge-flow measurements with a 1000 mm diameter rotor (DG-MECA, Gradignan, France; Charrier *et al.*, 2018). Since these measurements were performed on 1 m-long branches, the open-vessel artefact was avoided. Although intraspecific variation in xylem embolism resistance is generally rather limited (Lamy *et al.*, 2014; Schuldt *et al.*, 2016; Stojnić *et al.*, 2018; Jinagool *et al.*, 2018), the stem *P*_50_ of *L. nobilis* should be compared with caution to our microCT leaf *P*_50_ because plant material from both organs was from different individuals. The *L. nobilis* plants studied by Lamarque *et al.* (2018) included 2.5 m-tall branches from adult plants growing at the Domaine du Haut Carré on the campus of the University of Bordeaux (France).

### Anatomical measurements

Anatomical measurements using light and scanning electron microscopy were conducted at Ulm University. We randomly selected five, partially dried leaves from five branches that had previously been scanned at the SLS (*n*=5 leaves, or 3 branches). Transmission electron microscopy was based on freshly cut plant material (*n*=1 leaf, or 1 stem) from the same trees as those scanned at the SLS. Special attention was paid to select mature leaves and stems of similar size.

#### Scanning electron microscopy

Xylem segments of 5 mm in length were taken from branch and leaf midribs of the three species studied. For each species, we randomly selected three branches, and five leaves from five branches that were previously scanned at the SLS to obtain enough xylem tissue for our Scanning electron microscopy (SEM) measurements. These samples were dried overnight, mounted on SEM stubs, and sputter-coated with gold using a Balzers Union FL-9496 sputter device (Balzers, Liechtenstein). Micrographs of interconduit pits were obtained with a Phenom XL scanning electron microscope (Phenom World B.V., Netherlands) at an acceleration current of 5 kV. Bordered pit area (*A*_Pit_, µm^2^) and pit aperture area (*A*_Pit,Ap_, µm^2^) were measured for at least 100 pits per species. We calculated the pit aperture fraction (*F*_AP_) as the ratio of pit aperture area (*A*_Pit,Ap_) to pit border area (*A*_Pit_) (Lens *et al.*, 2011). These anatomical characters were measured with ImageJ (version 1.48v).

#### Light and transmission electron microscopy

Fresh branch segments of the same tree individual were collected on 8 October 2016 for transmission electron microscopy (TEM). For each species and organ (leaf midrib and branch), a single TEM sample was used. Small samples (*ca* 1 mm wide and 2 mm long) were prepared from both branches and leaf midribs, and fixed in standard fixative (2.5% glutaraldehyde, 0.1 mol phosphate, 1% saccharose, pH 7.3) overnight. After washing in phosphate buffer and post-fixation with 2% OsO_4_, samples were dehydrated with a propanol series (30%, 50%, 70%, 90%) and three treatments of 100% propanol. Samples were then immersed in 1.2-propylenoxide (VWR, Ulm, Germany) and gradually embedded in Epon resin (Sigma-Aldrich, Steinheim, Germany), which was polymerized at 60 °C over 48 h. Transverse, semi-thin sections were cut with an ultramicrotome (Leica Ultracut UCT, Leica Microsystems, Vienna, Austria), stained with 0.5% toluidine blue in 0.1 M phosphate buffer, and mounted on microscope slides using Eukitt.

The conduit area of branch and midrib xylem was measured on transverse sections using a Zeiss V16 AxioVision stereomicroscope (Oberkochen, Germany). Conduit area (A_C_, µm^2^) was based on at least 50 vessels per sample from various sections along the midrib and for three branches per species.

Ultra-thin sections (70–90 nm thick) were made with a Leica Ultracut UTC microtome (Leica Microsystems GmbH, Wetzlar, Germany) and placed on copper grids (Grids 300 mesh, Plano GmbH, Wetzlar, Germany). TEM observations were conducted using a JEOL 1400 TEM (JEOL, Tokyo, Japan) at 120 kV accelerating voltage and TEM images were taken with a digital camera (Soft Imaging System, Münster, Germany). For each sample, at least 10 different conduits with pit membranes were imaged, resulting in 15–25 individual pits. Only fully developed and clearly identifiable interconduit (i.e. intervessel or intertracheid) pit membranes were imaged. While most conduits in branch and midrib xylem were vessels, we cannot exclude that some conduits were tracheids. Pit membrane thickness was measured using ImageJ based on the mean value of three measurements along the membrane. For interconduit wall thickness (*T*_CW_), two measurements per conduit were taken at an area that was more than 500 nm away from a pit border to avoid potential variation in wall thickness near pit borders.

### Statistical analyses

Anatomical comparison between branches and leaves from the same specimen and between the three species was performed using Student’s *t*-test after testing for normal distribution (Sharpiro–Wilk test) and homogeneity of variance (Levene test). For the *P*_50_ values, differences between second or third order leaf veins and branch xylem, or between species, were considered significant when their 95% confidence intervals did not overlap (e.g. Duursma and Choat, 2017). We tested with Spearman’s rho for potential correlations between anatomical features and *P*_50_ values. Except where otherwise stated, all measurements were reported as mean ±standard error (SE). Statistical analyses were performed with SPSS Statistics 24.0.0.1 (IBM Corp., Armonk, NY, USA). Since our analyses were only concerned with properties of the data observed, they cannot be used to infer properties of a population.

## Results

### Xylem embolism resistance

**Fig. 1.**
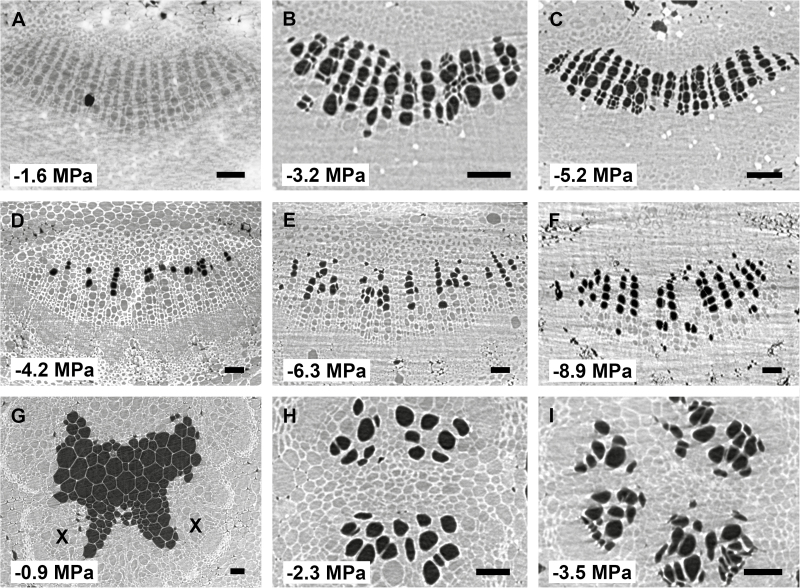
MicroCT slices showing cross sections of xylem tissue in leaf midribs of *Betula pendula* (A, B, C), *Laurus nobilis* (D, E, F), and *Liriodendron tulipifera* (G, H, I). Water-filled xylem conduits appear grey, while embolized conduits appear dark. The dark cells in the pith tissue of (G) do not represent embolized conduits. The corresponding water potential for each image is shown in the bottom left corner of each image. The adaxial side of all leaves is at the top of each image. X, xylem tissue. Scale bar: 50 µm for all images.

**Fig. 2.**
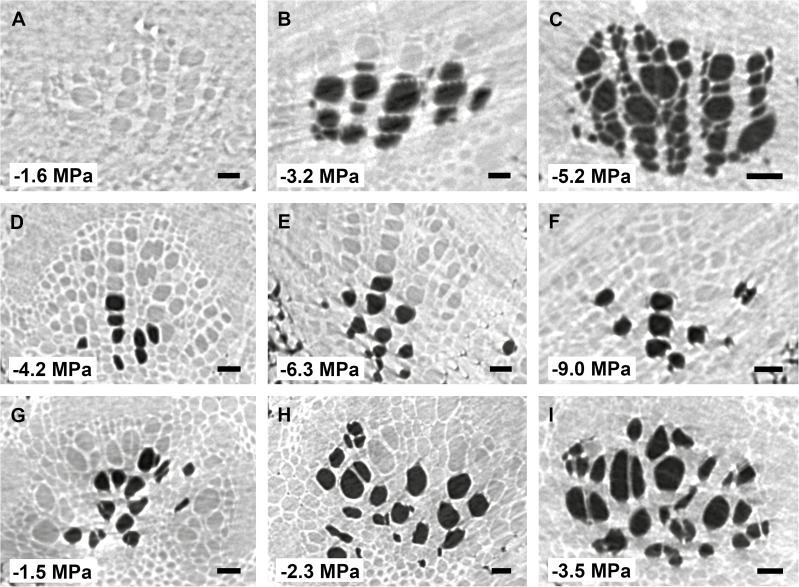
MicroCT slices showing cross sections of xylem conduits in second or third vein orders of *Betula pendula* (A, B, C), *Laurus nobilis* (D, E, F), and *Liriodendron tulipifera* (G, H, I), with reference to the corresponding xylem water potential measured. The exact vein order (second or third) could frequently not be determined based on microCT scans. The adaxial side of all leaves is at the top of each image. Scale bar: 20 µm for all images.

A total of 99 microCT scans were taken within the 72 h of beamtime, with leaves of the three species experiencing progressive levels of drought stress. Embolized conduits could easily be distinguished from functional, water-filled vessels in microCT scans due to the excellent phase contrast obtained: the lumina of functional vessels showed a grey appearance, while gas-filled vessels were considerably dark (Fig. 1). Secondary and third-order veins were frequently found near the midrib (Fig. 2). Observation of these veins along the 1.7 mm axial scan area was useful to find a connection between the vein and midrib, which frequently confirmed the status of the secondary vein order. In various cases, however, the scan area was too small to identify the vein connection, which made it impossible to distinguish secondary veins from third-order veins. As such, both second- and third-order veins were grouped together in our analyses.

**Fig. 3.**
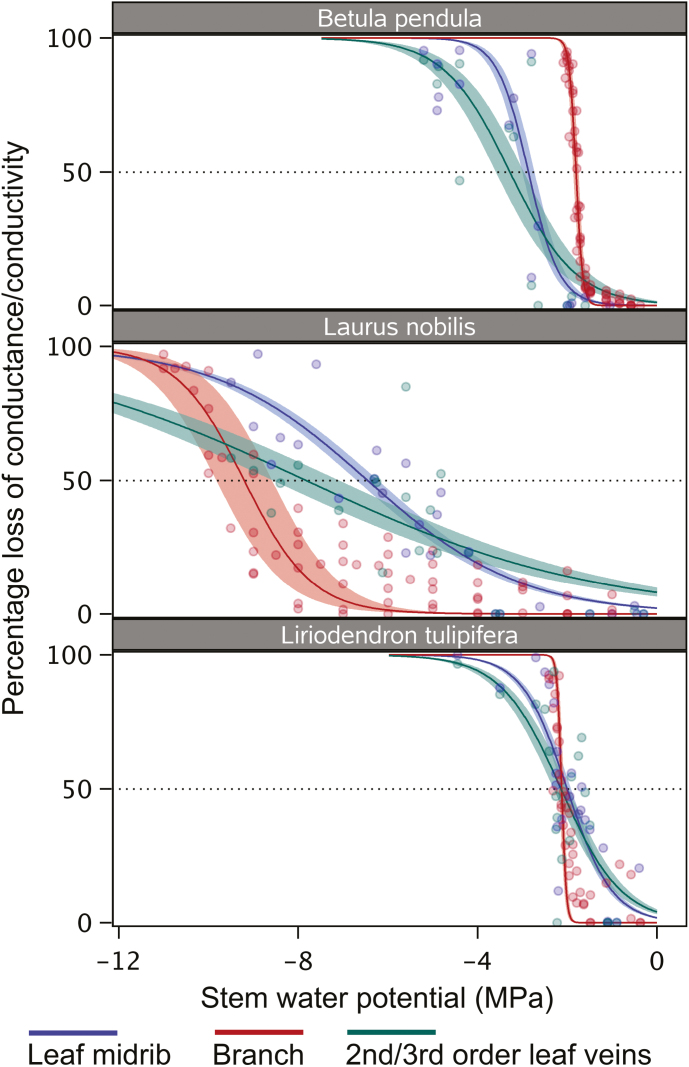
Vulnerability to drought-induced embolism for xylem tissue of different organs in three woody angiosperm species: *Betula pendula* (A), *Laurus nobilis* (B), and *Liriodendron tulipifera* (C). Percentage loss of conductance (PLC; leaves) or conductivity (branches) is plotted against stem water potential (MPa). Theoretical PLC values were determined based on microCT for leaf midribs and second or third vein orders, while hydraulic PLC values were based on a flow-centrifuge method for branches of *B. pendula* and *L. tulipifera*. Branch vulnerability curves were based on a flow-centrifuge method; stem data of *L. nobilis* were from Lamarque *et al.* (2018) and based on branches from different specimens than the one used for leaf sampling.

Cutting the veins within the scan area induced embolism artificially (Supplementary Fig. S1), and enabled us to quantify the amount of embolized conduits at a particular water potential before cutting (Figs 1, 2). The *P*_50_ values of leaf midribs were −2.01 ± 0.09 MPa for *L. tulipifera*, −2.87 ± 0.14 MPa for *B. pendula*, and −6.46 ± 0.24 MPa for *L. nobilis* (Fig. 1; Table 1; Supplementary Table S1). The vulnerability to embolism in midribs versus second- and third-order veins differed across species (Fig. 3; Table 1). For *L. tulipifera*, the difference of 0.13 MPa observed between the midrib and second or third vein order was not significant according to visual inspection of the 95% confidence intervals, but larger differences were found for *B. pendula* (0.41 MPa) and *L. nobilis* (1.37 MPa), with the midrib showing lower embolism resistance than the second and third vein orders (Fig. 3; Table 1). The slope of the vulnerability curve fitted for midribs and second- or third-order veins was rather similar for *B. pendula* and *L. tulipifera*, showing strong overlapping of the confidence bands (Fig. 3; Supplementary Table S1). However, the slope of the vulnerability curve for the second- and third-order veins of *L. nobilis* was less steep (7.75 ± 2.38) than the midrib (14.76 ± 2.24) (Fig. 3; Supplementary Table S1).

**Table 1.** Hydraulic vulnerability to embolism (*P*_50_, MPa) in leaf and stem xylem of *Betula pendula*, *Laurus nobilis*, and *Liriodendron tulipifera* based on X-ray computed tomography (leaf midrib, second and third vein order) and a flow-centrifuge method (branch)

Species	*P*_50_ (MPa)		
	X-ray microtomography		Centrifuge method
	Leaf midrib	Second and third vein orders	Branch
*Betula pendula*	−2.87 ± 0.14	−3.28 ± 0.31	−1.80 ± 0.01
*Laurus nobilis*	−6.46 ± 0.24	−7.83 ± 0.74	−9.21 ± 0.23^*a*^
*Liriodendron tulipifera*	−2.01 ± 0.09	−2.14 ± 0.17	−2.10 ± 0.02

All measurements were based on branches and leaves from a single specimen per species. Fitted value ±SE.

^*a*^ Based on Lamarque *et al.* (2018).

Branch embolism resistance based on the centrifuge flow method showed *P*_50_ values of −1.8 ± 0.01 MPa for *B. pendula* and −2.1 ± 0.02 MPa for *L. tulipifera* (Fig. 3; Table 1).

Embolism resistance between leaves and branches was more or less within the same range, although differences were observed across the three species studied. In *L. tulipifera*, vulnerability curves of the midrib, second- and third-order veins, and branch xylem overlapped in their confidence intervals, with their *P*_50_ values within a narrow range of −2.01 to −2.14 MPa (Fig. 3C). In *B. pendula*, confidence intervals of vulnerability curves of leaf and stem xylem did not overlap, with the leaf midrib *P*_50_ (−2.87 MPa) being 1 MPa more negative than stem *P*_50_ (−1.8 MPa) (Fig. 3A). The opposite situation was found for *L. nobilis*, with *P*_50_ values being more negative in branch xylem (−9.2 MPa) than second and third vein orders (−7.83 MPa), and the least negative *P*_50_ values were for the leaf midrib (−6.46 MPa).

### Xylem anatomy in relation to embolism resistance

Conduit area (*A*_C_) was wider in branches than in midribs of *B. pendula* and *L. tulipifera* (*P*=0.03 and *P*=0.01, respectively), whereas no significant difference was observed for *L. nobilis* (*P*=0.81; Table 2). In *B. pendula* and *L. nobilis*, the conduit wall thickness (*T*_CW_) was significantly thicker in branches than midribs (Table 2). Conduit wall thickness was 2.01 ± 0.32 µm in leaf xylem of *B. pendula* and 2.27 ± 0.46 µm in stem xylem (*P*=0.04). For *L. nobilis*, conduit walls were 3.62 ± 0.49 µm thick in leaf midribs and 4.48 ± 0.64 µm thick in stem xylem (*P*<0.01). No significant difference was observed in conduit wall thickness between stems and midribs for *L. tulipifera* (*P*=0.70).

**Table 2.** Wood anatomical features related to conduit and pit characteristics for xylem of branches and leaf midribs in *Betula pendula*, *Laurus nobilis*, and *Liriodendron tulipifera*

Species	Organ	*A*_C_ (µm^2^)	*A*_Pit_ (µm^2^)	*A*_Pit,Ap_ (µm^2^)	*F*_AP_ (%)	*T*_PM_ (nm)	*D*_PC_ (nm)	*T*_CW_ (µm)
*B. pendula*	Leaf midrib	96 ± 66*	4.54 ± 0.91*	1.28 ± 0.65*	0.35 ± 0.25*	240 ± 60*	405 ± 110*	2.01 ± 0.32*
	Branch	219 ± 115*	3.01 ± 0.86*	0.63 ± 0.13*	0.24 ± 0.08*	165 ± 27*	560 ± 131*	2.27 ± 0.46*
*L. nobilis*	Leaf midrib	231 ± 87	18.02 ± 2.81*	1.23 ± 0.18*	0.08 ± 0.03*	498 ± 68	410 ± 125	3.62 ± 0.49*
	Branch	278 ± 114	13.77 ± 3.81*	2.14 ± 1.24*	0.13 ± 0.09*	492 ± 125	411 ± 111	4.48 ± 0.64*
*L. tulipifera*	Leaf midrib	171 ± 33*	22.61 ± 7.06*	6.66 ± 3.01*	0.29 ± 0.07	305 ± 88	698 ± 248	3.54 ± 1.15*
	Branch	409 ± 158*	34.61 ± 8.82*	5.22 ± 2.24*	0.27 ± 0.07	268 ± 91	805 ± 87	2.99 ± 0.38*

*A*_C_, conduit area; *A*_Pit_, bordered pit area; *A*_Pit,Ap_, pit aperture area; *D*_PC_, pit chamber depth; *F*_AP_, pit aperture fraction; *T*_CW_, interconduit wall thickness; *T*_PM_, interconduit pit membrane thickness. Values are given as mean ±SD. *Significant differences between branches and midribs.

Significant differences were found in various pit characters between branch xylem and the xylem of leaf midribs (Table 2). The pit border surface area (*A*_Pit_) was larger in xylem of leaf midribs than branches for *B. pendula* and *L. nobilis*, but the opposite was found for *L. tulipifera*. Pit aperture surface areas (*A*_Pit,Ap_) were larger in the midrib than in the branches for *B. pendula* and *L. tulipifera*. *Laurus nobilis*, however, showed a larger mean pit aperture area in its branches than in the leaf midrib. The ratio between the pit aperture surface area and pit membrane surface area (*F*_AP_) was slightly higher in the leaf midrib than in branches for *B. pendula* and *L. tulipifera*, with values between 0.24 ± 0.08 and 0.35 ± 0.25. This indicates that the pit aperture area represents about 30% of the total pit border area for both species. *F*_AP_ values for *L. nobilis* were lower, with pit apertures occupying 8% of the pit border area in the midrib and 13% in branch xylem.

Pit chamber depth (*D*_PC_) was largest in *L. tulipifera* (698 and 805 nm for the midrib and branch xylem, respectively), followed by *B. pendula* (405 and 560 nm), and most shallow in *L. nobilis* (410 and 411 nm). These data suggest that pit borders in the leaf midrib were shallower than in branch xylem in the species studied, although this difference was only statistically significant for *B. pendula* (Table 2).

**Fig. 4.**
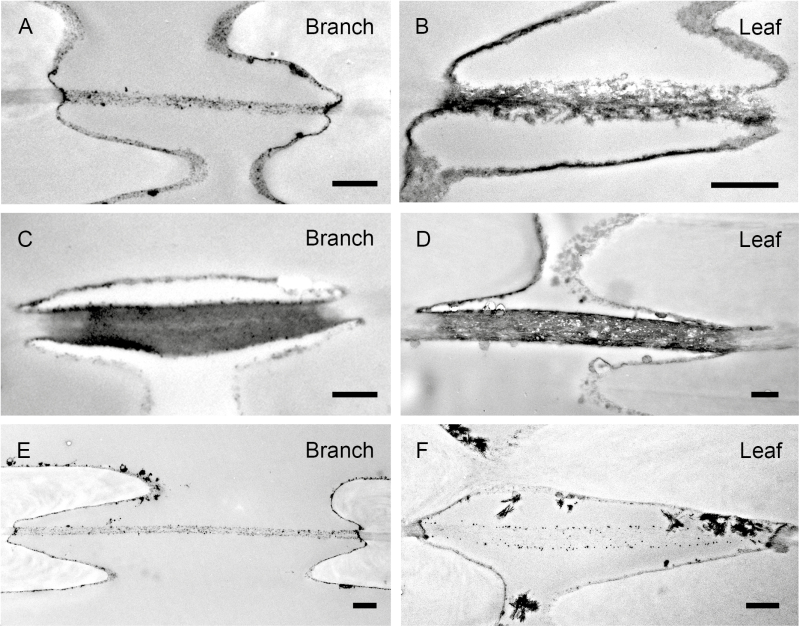
Transmission electron microscopy images of interconduit pit borders and pit membranes in *Betula pendula* (A, B), *Laurus nobilis* (C, D), and *Liriodendron tulipifera* (E, F) from branch xylem (A, C, E) and leaf midribs (B, D, F). Pit apertures are (partly) visible when the section was cut through the centre of the pit border (A, C, D, E). Scale bars: 500 nm.

The pit membranes between neighbouring conduits showed a general similarity in ultrastructure between leaves and branches (Fig. 4). In all species and organs, pit membranes included dark (i.e. highly electron dense) particles, which were typically largest near the outermost layers of the pit membrane (Fig. 4F). Pit membranes of *L. nobilis* had an equally dark appearance in xylem of the branch and midrib (Fig. 4C, D), while pit membranes were considerably darker in midribs than in branches of *B. pendula* (Fig. 4A, B). Pit membranes in *L. tulipifera* (Fig. 4E, F) were most transparent, showing a smaller amount of dark nanoparticles after treatment with OsO_4_ than *B. pendula* and *L. nobilis*.

**Fig. 5.**
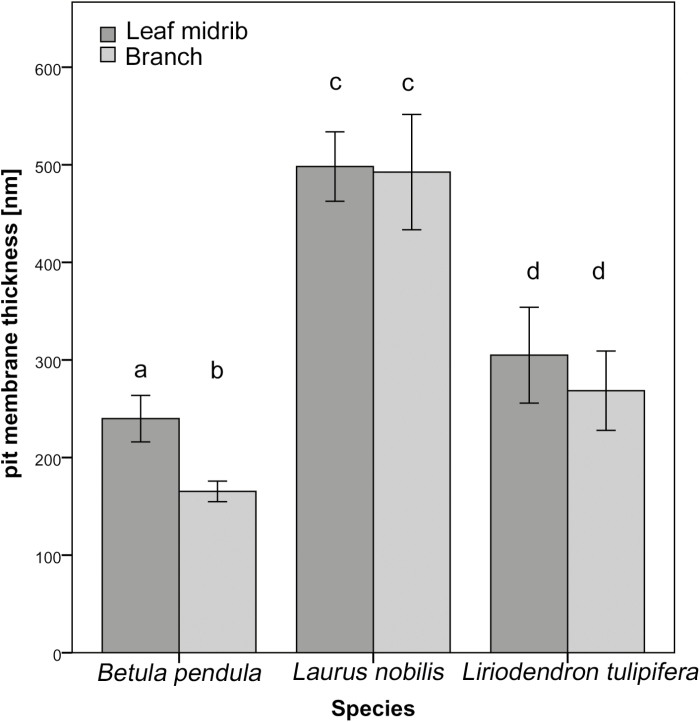
Comparison of interconduit pit membrane thickness (nm) for xylem of leaf midribs (dark grey bars) and branches (light grey bars) in *Betula pendula*, *Laurus nobilis*, and *Liriodendron tulipifera*. The bars show mean values, and the error bars the standard errors. Different letters indicate intraspecific and interspecific statistical significance.

Values of mean pit membrane thickness (*T*_PM_) were highest in *L. nobilis*, where they were close to 500 nm in the midrib and stem xylem, while thinner interconduit pit membranes occurred in *L. tulipifera* (305 and 268 nm for midribs and branches, respectively) and *B. pendula* (Table 2; Figs 4, 5). The thinnest pit membranes were observed in *B. pendula*, which was the only species showing significant difference in pit membrane thickness between the leaf midribs (240 ± 60 nm) and branches (165 ± 27 nm) (Fig. 4A, B). This significant difference in *T*_PM_ for *B. pendula* was linked to a 1 MPa difference in *P*_50_ values for both organs: the thickest pit membranes (240 ± 60 nm) in the midrib corresponded to a more negative *P*_50_ value (−2.87 ± 0.14 MPa), while the thinner pit membranes (165 ± 27 nm) corresponded to a branch xylem *P*_50_ of −1.8 ± 0.01 MPa. When analysing *T*_PM_ and *P*_50_ values for the three species, including branches and midribs, a significant negative correlation was found (*r*=−0.92, *n*=6, *P*<0.01).

## Discussion

### The hydraulic vulnerability segmentation hypothesis

Our microCT observations show that leaf xylem of the three angiosperm species studied is fairly resistant to embolism, with *P*_50_ values around −2 MPa or less negative (Fig. 3; Table 1). High embolism resistance was especially found in leaf veins of *L. nobilis*, despite the fact that all microCT scans were performed on cut branches, which may underestimate embolism resistance due to artificial air entry. The relatively high resistance to xylem embolism observed agrees with recent studies based on direct, non-invasive methods, such as the optical method (Brodribb *et al.*, 2016a, b; Rodriguez-Dominguez *et al.*, 2018; Lamarque *et al.*, 2018) and earlier microCT observations of leaves (Bouche *et al.*, 2016*a*; Skelton *et al.*, 2017; Scoffoni *et al.*, 2017*a*). Moreover, minor variation in xylem embolism resistance within a leaf may occur, because second- and third-order veins were found to show an equal or even higher embolism resistance than the midrib, as suggested previously (Brodribb *et al.*, 2007; Scoffoni *et al.*, 2017*b*).

Comparing xylem embolism resistance of leaves with stems was feasible because non-xylem leaf tissue was excluded in our microCT analyses. While the major advantage of microCT observations is direct, visual observation of embolism without artefacts, which may be introduced by manipulating a hydraulic system under negative pressure, the drawback of this method is that hydraulic conductance cannot be measured directly (Jansen *et al.*, 2015). Instead, vulnerability curves based on microCT rely either on the assumption that all water-filled conduits are functional, or on the difference in filled versus embolized conduits before and after cutting the scanned tissue (Supplementary Fig. S1). There is, however, convincing evidence that microCT observations agree well with hydraulic measurements for various species (Choat *et al.*, 2010, 2016; Bouche *et al.*, 2016*b*; Nolf *et al.*, 2017; Torres-Ruiz *et al.*, 2017; Charrier *et al.*, 2018; Lamarque *et al.*, 2018). Alternative, indirect methods to determine xylem vulnerability to embolism in leaves should be interpreted with caution, because no direct comparison can be made between microCT observations (excluding the non-xylem tissue) and methods that estimate whole-leaf responses (i.e. including xylem *and* non-xylem tissue) to the decline in leaf hydraulic conductance (Bouche *et al.*, 2016*a*; Trifiló *et al.*, 2016; Scoffoni *et al.*, 2017*b*; Scoffoni and Sack, 2017). Leaves of *L. tulipifera*, for instance, were found to have a 50% reduction of the whole-leaf hydraulic conductance at −1.2 MPa based on the rehydration kinetics method (Johnson *et al.*, 2012; 2016). Therefore, the xylem tissue seems to be more resistant to dysfunction than the outer xylem tissue, which provides the most important bottleneck causing a decline of leaf hydraulic conductance (Scoffoni *et al.*, 2017*b*; Scoffoni and Sack, 2017).

A major result of this paper is that xylem embolism resistance is more or less similar between leaves and stems. This finding was also supported by similarity in bordered pit membrane thickness, which is a useful determinant of drought-induced embolism resistance (Li *et al.*, 2016). In particular, we found similar embolism resistance of xylem between leaves and branches for *L. tulipifera*. A previously published *P*_50_ value of −2.99 MPa for stems of *L. tulipifera* would indicate higher embolism resistance of stems based on the air-injection method (Johnson *et al.*, 2011), but could also reflect intraspecific variation and/or methodological artefacts (Yin and Cai, 2018). A branch *P*_50_ value for seedlings of *L. tulipifera* based on microCT was −1.61 MPa (data not shown), which is slightly less negative than the *P*_50_ values for leaves and stems reported here (Table 3), suggesting that leaf xylem of this species is not more vulnerable to embolism than are branches.

An even higher embolism resistance for leaves than branches was found for *B. pendula*, which is the opposite of what would be predicted based on the hydraulic vulnerability segmentation hypothesis. Previously published *P*_50_ values for branches of *B. pendula* were in good agreement with our centrifuge-flow measurements, with values between −1.8 MPa and −2.3 MPa (Cochard *et al.*, 2005; González-Muñoz *et al.*, 2018). Moreover, the thicker pit membranes in leaves than in branches (Fig. 5) are in line with their high leaf embolism resistance.

Lower xylem embolism resistance in leaves than branches was found for *Vitis vinifera* (Charrier *et al.*, 2016; Hochberg *et al.*, 2016) and olive (Rodriguez-Dominguez *et al.*, 2018), and could be suggested for *L. nobilis*, although our data should be interpreted with caution because of the use of different specimens. Stem *P*_50_ values of *L. nobilis* reported in literature are highly variable, ranging from −1.5 to −3.5 MPa (Salleo and Lo Gullo, 1993; Salleo *et al.*, 2009; Nardini *et al.*, 2017), but these values may underestimate embolism resistance due to artefacts (Cochard *et al.*, 2015). The leaf *P*_50_ values obtained in this study for *L. nobilis* (−6.46 and −7.83 MPa for the midrib and second-/third-order veins, respectively) are much more negative than these earlier records, but within the same order of magnitude as the −7.94 to −9.21 MPa values based on three different methods: (i) microCT of stems from intact plants, (ii) the optical method of leaf veins, and (iii) the centrifuge-flow method on 1 m-long stem segments (Lamarque *et al.*, 2018). While the latter authors showed that embolism resistance was not different between stem and leaf veins of *L. nobilis* when using samples from the same plant, embolism resistance of leaves based on microCT could be interpreted as being equally vulnerable to that of stems.

The relatively high embolism resistance observed in leaves does not imply that hydraulic segmentation may not occur without any difference in xylem embolism resistance. First, even if the stem and leaf xylem show equal resistance to embolism, or if the leaves show a higher resistance than the stems, leaves can still provide hydraulic segmentation because the outer xylem tissue (i.e. the non-vascular leaf tissue) can act as a hydraulic bottleneck in the system, protecting the xylem in the vascular bundles (Scoffoni *et al.*, 2017*a*). In this way, leaf xylem would be less expendable as outside xylem, which may recover more easily from drought-induced decline due to aquaporin activity (Maurel *et al.*, 2016; Groszmann *et al.*, 2017). Recovery of embolized xylem conduits in leaves, on the other hand, shows physical constraints and is unlikely to represent a dynamic and fast process on a daily basis (e.g. Mayr *et al.*, 2014; Bouche *et al.*, 2016*a*). Second, leaves can be at more negative water potentials than stems via transpiration and/or cuticular conductance. Therefore, leaves may still experience xylem embolism before stems, creating a hydraulic fuse for stem xylem (Tyree and Ewers, 1991; Wolfe *et al.*, 2016). Stem and leaf xylem water potential measurements would be required to estimate hydraulic safety margins (defined as the most minimum xylem water potential experienced by a plant in the field minus the *P*_50_ value of a tissue; Meinzer *et al.*, 2009; Choat *et al.*, 2012) and to put the findings of this paper in an ecological context. Based on the minimum xylem water potentials reported in literature, which are −1.19 for *L. tulipifera* (Johnson *et al.*, 2012), −2.64 for *B. pendula* (Cochard *et al.*, 2005), and −4.2 MPa for *L. nobilis* (Lamarque *et al.*, 2018), hydraulic safety margins would be positive for *L. nobilis* and *L. tulipifera*, except for branches of *B. pendula*. A positive hydraulic safety margin would indicate that embolism levels corresponding to *P*_50_ are unlikely for branches and leaves in the field, while a negative hydraulic safety margin for *B. pendula* would suggest that embolism in the field is slightly higher for this species. The lab-based data presented in this paper, however, do not allow us to make any firm statement about embolism occurrence in the field.

Moreover, future work should focus on whole plant modelling that takes into account the vulnerability of each organ to embolism to better understand the degree of ‘true’ segmentation across species. While it is clear that xylem conduits form a morphological and developmental continuum over long distances, from the roots to the minor leaf veins, anatomical bottlenecks are well known to occur at nodes, bifurcations, and stem–petiole abscission zones, making conduit connections non-randomly distributed and potentially segmented (Salleo *et al.*, 1984; André *et al.*, 1999; André, 2005; Rančić *et al.*, 2010; Wolfe *et al.*, 2016).

### Embolism resistance in relation to xylem anatomy

Additional support for relatively high embolism resistance in leaves is provided by the interconduit pit membrane thickness, which was similar between both organs in *L. nobilis* and *L. tulipifera*, but significantly higher in leaves than in branches for *B. pendula*. Thick pit membranes could provide higher hydraulic safety due to narrow pore volumes and/or long pore pathways, which would increase the number of constrictions within this pathway and therefore bubble snap-off (Roof, 1970; Kovscek *et al.*, 2007; Schenk *et al.*, 2017). The difference in pit membrane thickness between leaves and stems of *B. pendula* may explain the 1 MPa difference in *P*_50_ between branches and midribs of this species. While intervessel pit membrane thickness was found to be a determinant of *P*_50_ (Li *et al.*, 2016), these findings also suggest that variation in pit membrane thickness does not always correspond to considerable changes in *P*_50_, at least not among organs within a plant. Given the differences in conduit size between leaves and stems, especially in *L. tulipifera* and *B. pendula*, our findings also suggest that pit membrane thickness and the associated resistance to air-seeding is not affected by conduit size, which would be expected based on the assumption that wide conduits are more vulnerable to xylem embolism than narrow ones (Hargrave *et al.*, 1994; Sperry *et al.*, 1994; Tyree and Zimmermann, 2002).

To our knowledge, our TEM observations of the midrib xylem are the first to show that post-fixation with OsO_4_ results in a dark staining of interconduit pit membranes in leaves, similar to what has been found for stems and branches (Schmid and Machado, 1968; Jansen *et al.*, 2009; Schenk *et al.*, 2017, 2018; Zhang *et al.*, 2017). This staining reaction is most likely caused by binding of osmium particles to double carbon bonds in unsaturated fatty acid chains of lipids (Riemersma, 1968; Schenk *et al.*, 2017, 2018). More research of xylem tissue across different organs is needed to address the functional role of lipid surfactants in pit membranes and their potential effect on air-seeding (Jansen and Schenk, 2015; Schenk *et al.*, 2015, 2017, 2018; Jansen *et al.*, 2018).

Despite the limited number of species studied, some anatomical features could explain the high embolism resistance in *L. nobilis*. Compared with *B. pendula* and *L. tulipifera*, *L. nobilis* showed the thickest interconduit walls (*T*_CW_), thickest interconduit pit membranes (*T*_PM_), most shallow pit chambers (*D*_PC_), and a low pit aperture fraction (*F*_AP_, i.e. the ratio between the pit aperture surface area to the pit border surface area). Thick intervessel walls can be suggested to increase hydraulic safety by avoiding wall collapse under negative pressure (Hacke *et al.*, 2001), while shallow pit chambers and a low pit aperture fraction could provide mechanical support for aspirated pit membranes, resulting in increased air-seeding pressures (Choat *et al.*, 2008; Lens *et al.*, 2011). The relatively low (8–13%) pit aperture fraction of *L. nobilis* had a similar order of magnitude to the pit aperture fractions reported in *Acer* (Lens *et al.*, 2011), but more data on additional species would be needed to provide a functional explanation of this feature.

## Conclusions

In summary, the findings of this paper raise questions about the assumption that leaf xylem is the main driving mechanism behind the hydraulic vulnerability segmentation hypothesis, because embolism resistance in leaves and stems can be similar (*L. tulipifera*), possibly higher in leaves than stems (*B. pendula*), or possibly lower in leaves than stems (*L. nobilis*). Further work is required to test whether the underlying mechanism behind hydraulic segmentation between plant organs is species-specific across a wide range of angiosperms, and to what extent there is intraspecific variation. We recommend that sufficient care should be taken in distinguishing xylem embolism resistance in leaf veins from whole-leaf hydraulic measurements. Finally, anatomical features such as interconduit pit membranes may provide additional evidence for embolism resistance and could improve our poor understanding of the mechanisms behind air-seeding. More detailed anatomical observations are also needed to investigate whether anatomical segmentation or compartmentalization occurs at the root to the stem transition, at the stem–petiole tissue level, and/or along different vein orders or vein connections (Cochard *et al.*, 1992; Hacke and Sauter, 1996).

## Supplementary data

Supplementary data are available at *JXB* online.

Fig. S1. MicroCT slices showing cross sections of xylem tissue in leaf midribs of *Betula pendula*, *Laurus nobilis*, and *Liriodendron tulipifera*.

Fig. S2. Three dimensional reconstruction based on microCT showing a cross section through a leaf of *Liriodendron tulipifera* at −2.3 MPa.

Table S1. Statistical comparison of the theoretical hydraulic loss of conductance between different organs and species.

Supplementary Material
