# Supplementary material for: Is xylem of angiosperm leaves less resistant to embolism than branches? Insights from microCT, hydraulics, and anatomy
Source: J Exp Bot. 2018 Sep 1;69(22):5611–23. doi: 10.1093/jxb/ery321 (PMC6255699; doi:10.1093/jxb/ery321)
Supplement: Supplementary Material [file ery321_suppl_supplementary_figures_tables.pdf]

Supplementary Tables

Supplementary Table 1: The theoretical hydraulic loss of conductance (leaf midribs and 2nd / 3rd vein orders) or conductivity (branches) estimated for the angiosperm species *Betula pendula*, *Laurus nobilis*, and *Liriodendron tulipifera* based on microCT. The underlying model is given by f = 100 / (1 + exp ((S / 25) * (x - P_{50})), where "S" is the slope of the function, and P_{50} the water potential corresponding to 50% loss of hydraulic conductance / conductivity. Values are mean ± standard error.

Species	Organ	S	P-value for S	P_{50} (MPa)	P-value for P_{50}	R^2 for the model	P-value for the model
B. pendula	Branch	280.03 ± 24.76	< 0.01	-1.80	< 0.01	0.93	< 0.01
	Midrib	71.80 ± 31.33	0.04	-2.87	< 0.01	0.78	< 0.01
	2nd and 3rd vein orders	34.00 ± 13.61	0.03	-3.28	< 0.01	0.67	< 0.01
L. nobilis	Midrib	14.76 ± 2.24	< 0.01	-6.46	< 0.01	0.84	< 0.01
	2nd and 3rd vein orders	7.75 ± 2.38	< 0.01	-7.83	< 0.01	0.50	< 0.01
L. tulipifera	Branch	155.47 ± 18.34	< 0.01	-2.10	< 0.01	0.86	< 0.01
	Midrib	49.71 ± 11.51	< 0.01	-2.01	< 0.01	0.71	< 0.01
	2nd and 3rd vein orders	37.14 ± 13.96	0.02	-2.14	< 0.01	0.51	< 0.01

Supplementary figures

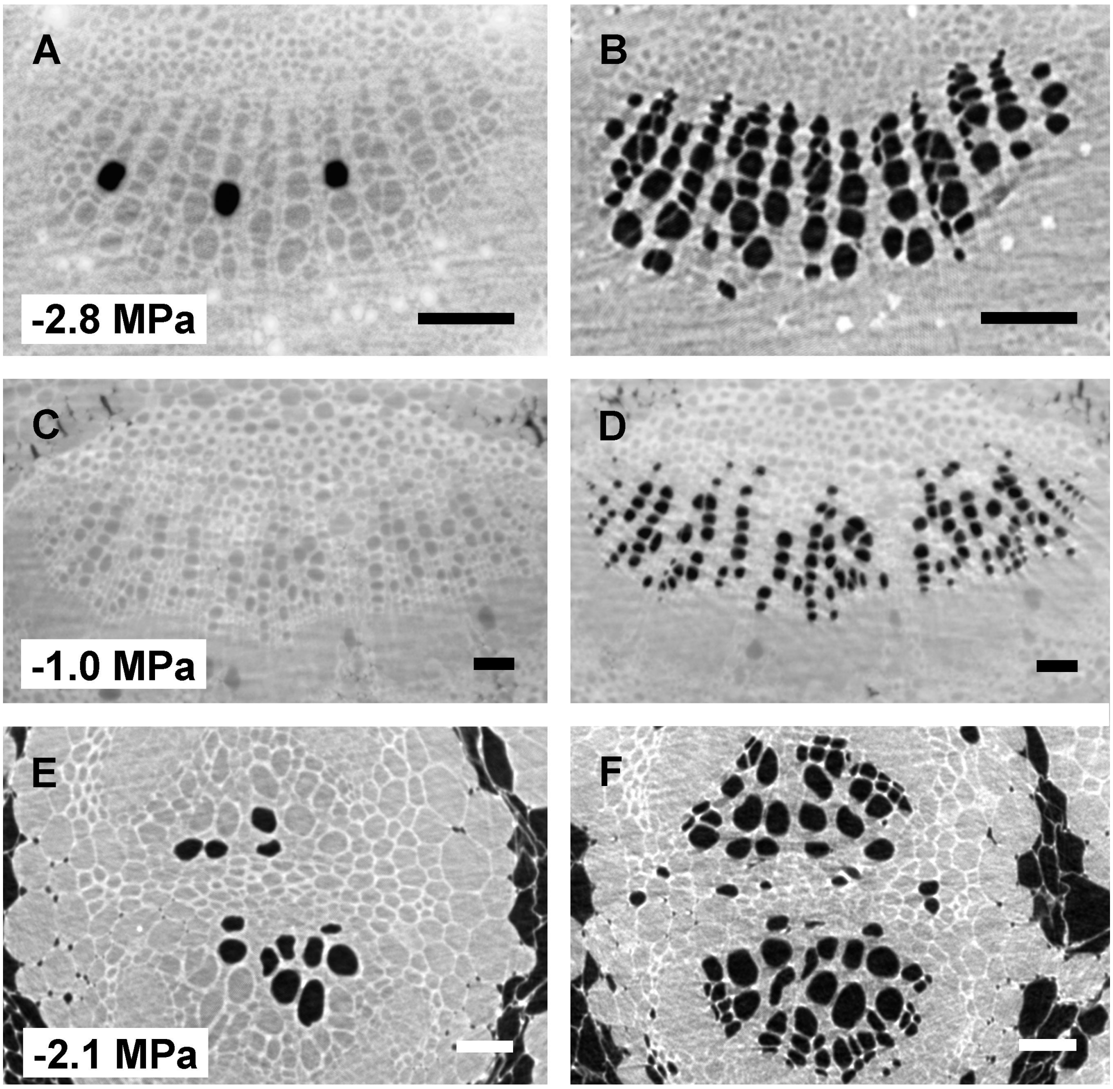

Supplementary Figure 1: MicroCT slices showing cross sections of xylem tissue in leaf midribs of *Betula pendula* (A, B), *Laurus nobilis* (C, D), and *Liriodendron tulipifera* (E, F). The images on the left (A, C, E) were taken at a xylem water potential as indicated for each image. After cutting the midrib with a sharp razor blade to induce embolism artificially, the same midrib was imaged at the cut end (B, D, F), and corresponding slices before and after cutting were used to estimate the total number and cross-sectional area of all water conducting cells, as well as the relative amount of embolism before cutting the midrib. The adaxial side of all leaves is at the top of each image. Scale bars = 50 µm.

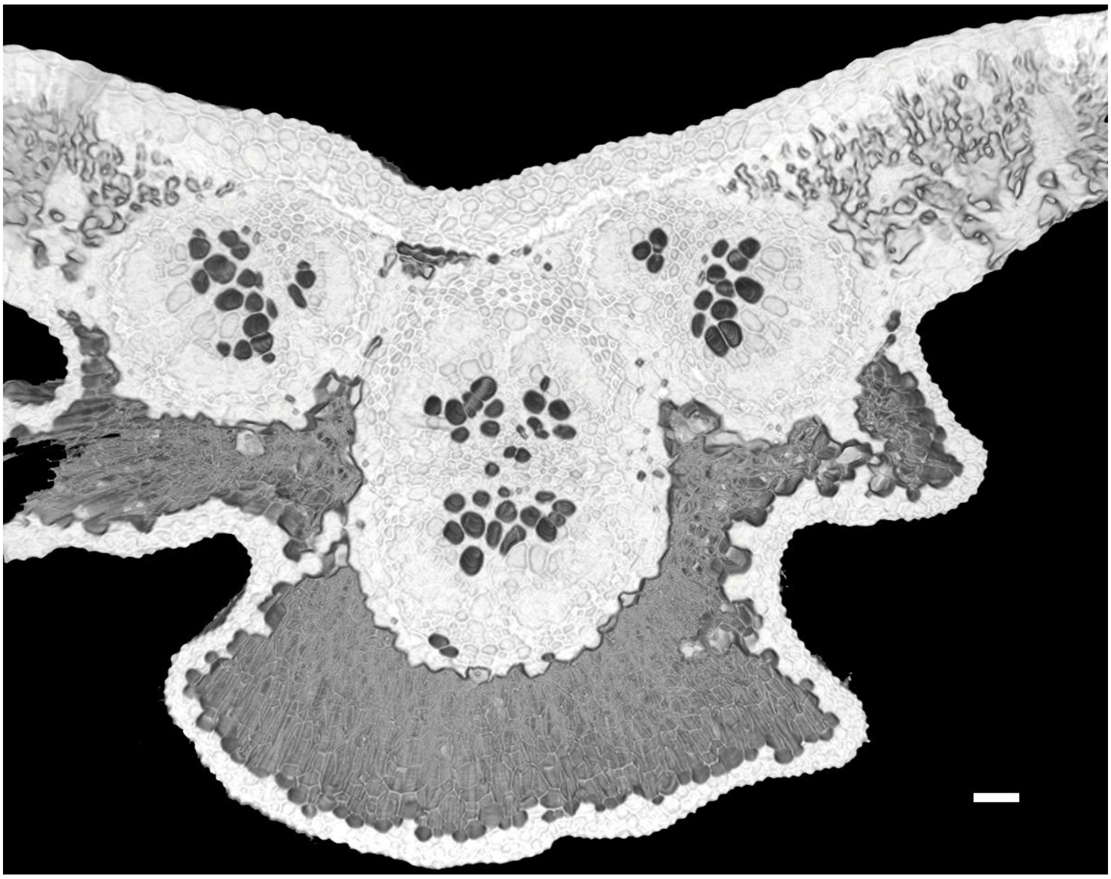

Supplementary Figure 2: Three dimensional reconstruction based on microCT showing a cross section through a leaf of *Liriodendron tulipifera* at -2.3 MPa. The water-filled conduits in the xylem tissue of the midrib (centre) and secondary veins (upper right and left) are grey, while embolised conduits are dark. Scale bar = 50 µm.
